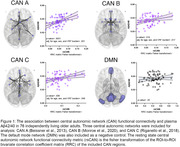# Central Autonomic Dysfunction and Plasma Ab_42/40_

**DOI:** 10.1002/alz.089443

**Published:** 2025-01-09

**Authors:** Trevor Lohman, Arunima Kapoor, Allison C Engstrom, Fatemah Shenasa, Amy Nguyen, John Paul M Alitin, Aimée Gaubert, Kathleen E. Rodgers, David Bradford, Mara Mather, Duke Han, Elizabeth Head, Julian F Thayer, Daniel A. Nation

**Affiliations:** ^1^ University of Southern California, Los Angeles, CA USA; ^2^ University of California, Irvine, Irvine, CA USA; ^3^ University of Southern California, Leonard Davis School of Gerontology, Los Angeles, CA USA; ^4^ University of Arizona, Tucson, AZ USA; ^5^ The UC Irvine Institute for Memory Impairments and Neurological Disorders (UCI MIND), Irvine, CA USA; ^6^ Keck School of Medicine, Los Angeles, CA USA

## Abstract

**Background:**

Higher order regulation of autonomic function is maintained by cortical and subcortical interconnected regions within the brain, collectively referred to as the central autonomic network (CAN) (Benarroch, 1993). Despite the well‐established relationship between autonomic dysfunction and AD (Femminella et al., 2014) the relationship between CAN functional connectivity and biomarkers of AD, such as Ab_42/40_ ratio, remains unexplored.

**Methods:**

76 independently living older adults were recruited from the community to undergo brain fMRI and venipuncture. Study exclusions were history of clinical stroke, dementia, major neurological or psychiatric disorder, current organ failure or other uncontrolled systemic illness. Resting state fMRI data were acquired and analyzed to quantify CAN functional connectivity from 3 previously described CAN networks with the CONN Functional Toolbox (Nieto‐Castanon, 2020). Default mode network connectivity was also included for analysis as a negative control. Plasma Aβ_40_ and Aβ_42_ concentrations were obtained by digital immunoassay with the Simoa Neurology 3‐Plex A (N3PA) Advantage Kit (Quanterix). Vascular risk factors were evaluated through interviews with the participant and informant, and included history of cardiovascular disease, hypertension, hyperlipidemia, type 2 diabetes, atrial fibrillation, and transient ischemic attack. The relationship between CAN functional connectivity and plasma Ab_42/40_ ratio was compared using linear regression adjusting for demographic covariates and vascular risk factor burden.

**Results:**

CAN functional connectivity was positively associated with Ab_42/40_ for all three CAN models (CAN A (Beissner et al., 2013) P= .0001; CAN B (Monroe et al., 2020) P= .018; CAN C (Riganello et al., 2018) P= .006), and remained so after adjustment for age, sex, and vascular risk factor burden. Default mode network connectivity was not significantly associated with Ab_42/40_ (Figure 1).

**Conclusion:**

Older adults with decreased central autonomic network connectivity exhibit lower plasma Ab_42/40_, indicating greater cerebral Ab_1‐42_ retention. This correlation could not be accounted for by age, sex, or vascular risk factor burden, suggesting a direct relationship between central autonomic function and cerebral amyloidosis. Further studies should explore the implications of CAN dysfunction for autonomic changes during the early stages of AD pathophysiology.